# Ocular injuries in padel: findings from a survey on frequency, risk factors, and perceptions toward protective eyewear

**DOI:** 10.1038/s41433-026-04447-8

**Published:** 2026-04-20

**Authors:** Francesco Aiello, Gabriele Gallo Afflitto, Alessio Martucci, Giulio Pocobelli, Massimo Cesareo, Vincenzo Maurino, EyePad Research Group, Francesco Aiello, Gabriele Gallo Afflitto, Alessio Martucci, Giulio Pocobelli, Massimo Cesareo, Vincenzo Maurino, Francesca Ceccarelli, Andrea Di Vossoli, Lorenzo Fabozzi, Giulia Fioravanti, Giulia Franceschini, Daniele Gaudenzi, Rodolfo Mastropasqua, Francesco Matarazzo, Luigi Mosca, Lorenzo Motta, Filomena Palmieri, Vito Romano, Domenico Schiano Lomoriello, Pier Luigi Surico, Carlo Nucci, Carlo Nucci

**Affiliations:** 1 Ophthalmology Unit, Department of Experimental Medicine, University of Rome “Tor Vergata”, Rome, Italy; 2 Moorfields Eye Hospital NHS Foundation Trust, London, UK; 3 Southend Hospital Eye Unit, Southend University Hospital, Southend-on-Sea, UK; 4 University of Rome “Tor Vergata”, Rome, Italy; 5 University Hospitals Sussex NHS Foundation Trust, Brighton and Hove, UK; 6 Centro Oculistico Chirurgico Fioravanti, Bologna, Italy; 7 UOSD Cardio-Thoracic Anaesthesia and Intensive Care Unit, University of Rome “Tor Vergata”, Rome, Italy; 8 San Martino Hospital, Section of Ophthalmology, Genova, Italy; 9 Department of Neuroscience, Imaging and Clinical Sciences, University G. D’Annunzio Chieti-Pescara, Chieti, Italy; 10 Department of Physics “Ettore Pancini”, University of Naples “Federico II”, 80131 Naples, Italy; 11 Department of Ophthalmology, Agostino Gemelli University Polyclinic Foundation - IRCCS, Rome, Italy; 12 Department of Neuroscience, Eye Unit, University of Padova, Padova, Italy; 13 Eye Unit, Department of Medical and Surgical Specialties, Radiological Sciences and Public Health, University of Brescia, Brescia, Italy; 14 IRCCS Bietti Foundation, Rome, Italy; 15 Department of Organs of Sense, University of Rome La Sapienza, Rome, Italy

**Keywords:** Epidemiology, Risk factors, Trauma

## Abstract

**Objectives:**

To characterize the proportion of respondents reporting padel-related ocular injury and its perceived long-term consequences, identify independent factors associated with injury reporting, and assess players’ acceptance of protective eyewear.

**Methods:**

A prospective, cross-sectional, voluntary online survey was distributed nationally via Italian padel websites and social-media forums between April 2024 and March 2025. A total of 583 respondents formed the analytic cohort. The 31-item questionnaire captured demographics, playing habits, ocular injury history, perceived impact on quality of life (QoL), vision (QoV), work, and attitudes toward protective goggles. Responses were limited to one submission per personal email account. Univariate and multivariate logistic regression analyses were performed to determine predictors of ocular trauma.

**Results:**

Sixty players (10.3%) self-reported at least one ocular injury; 55 (92%) were attributed to ball impact. Among injured participants, 25% described long-lasting reductions in QoL, 15% in QoV, and 8.3% in work performance; 6 players (10%) reported undergoing ocular surgery. Weekly playing frequency emerged as the only independent factor associated with injury reporting: compared with <1 match/week, the odds of injury peaked at 4–6 matches/week (OR 26.85; *p* < 0.001). Only 56 respondents (9.6%) strongly endorsed protective goggles use.

**Conclusion:**

Among respondents to this convenience-based survey, 10.3% reported having sustained ocular trauma, with up to one quarter of injured individuals describing long-lasting sequelae. Acceptance of protective goggles was low. These findings may inform targeted educational initiatives and the development of sport-specific safety recommendations for this rapidly expanding sport.

## Introduction

Padel is a rapidly growing racket sport that combines elements of tennis and squash and is characterized by high-speed exchanges, enclosed court play, and frequent close-range interactions between players [1]. Its accessibility, social nature, and relatively low entry barrier have contributed to its widespread adoption in both amateur and competitive settings [1, 2]. While the physical benefits of padel are well documented, increasing participation has raised concerns regarding the risk of sport-related injuries, particularly in the context of high-velocity impacts with balls, rackets, or surrounding structures [1, 3, 4].

Among the spectrum of injuries associated with padel, ocular trauma represents a potentially vision-threatening yet underreported category. Sports-related eye injuries have been widely studied in disciplines such as squash [5] and tennis [5]. as well as in other high-impact sports including basketball [6]. A recent systematic review of racquet sports reported that the vast majority of ocular injuries occur in the absence of certified protective eyewear, highlighting the preventive potential of appropriately designed eye protection [5] In parallel, contemporary emergency department data demonstrate that ocular injuries related to racquet and paddle sports are increasing as participation rises; for example, a cross-sectional analysis of the U.S. National Electronic Injury Surveillance System documented a marked increase in pickleball-related eye injuries between 2005 and 2024, most commonly due to ball impact [7, 8].

Although specific epidemiological data on ocular trauma in padel remain lacking, the sport’s structural and technical characteristics suggest biological plausibility for similar injury mechanisms. Official regulations define a 10 m × 20 m fully enclosed court, substantially smaller than a standard tennis court, and incorporate rebound walls that sustain play within confined boundaries [9]. The padel ball is comparable in size and mass to a tennis ball, supporting similar impact dynamics, yet the reduced playing distance and frequent close-range exchanges may increase the likelihood of short-range impacts. By comparison, squash, which is played in an even more confined indoor court, has historically been associated with a recognized burden of ocular trauma [10]. Together, these comparisons support the rationale for investigating eye injuries specifically within the padel context. Notably, formal requirements for protective eyewear are not currently specified within International Padel Federation rules, and any proposed protective device in Europe would need to comply with the applicable personal protective equipment regulatory framework [9].

In light of the absence of sport-specific data, there is a clear need to investigate the burden and clinical relevance of ocular injuries in padel, as well as players’ awareness and attitudes toward preventive strategies.

The primary aim of this study was to characterize the proportion of respondents reporting ocular trauma, as well as the reported mechanisms and perceived clinical consequences. Secondary objectives included identifying demographic and sport-related risk factors associated with injury occurrence and evaluating participants’ perceptions regarding the use of protective eyewear. To address these aims, we conducted a voluntary, convenience-sample, web-based survey targeting individuals engaged in padel practice across Italy.

## Materials and methods

This cross-sectional study using survey methodology was approved by the Regional Review Board of Lazio Area 2 (ID: 90.24 CET2 ptv_utv) and conducted in accordance with the ethical principles outlined in the Declaration of Helsinki. Informed consent was implied by voluntary completion of the survey, as indicated in the introductory section of the survey. All data collection and management procedures adhered to applicable provisions of the Health Insurance Portability and Accountability Act (HIPAA). The questionnaire was reviewed and evaluated by an epidemiologist and biostatistician from the University of Rome “Tor Vergata”. Prior to full dissemination, the questionnaire was pilot-tested in the first 30 respondents to assess clarity and comprehensibility; minor wording adjustments were implemented to improve understanding, and these pilot responses were excluded from the final analysis.

The survey was developed and disseminated using the Google Forms platform and was accessible online from April 2024 to March 2025. To ensure that each participant could submit only one response, access required users to log in with a personal email account. However, no email addresses were collected or stored at any stage, in full compliance with data protection and privacy regulations.

This survey employing conditional branching (Supplementary File 1) comprised 31 items, including both multiple-choice and free-text response formats, and was organized into five thematic domains: [1] demographic and geographic characteristics (e.g., age, gender, region, and city of residence) [2] ocular health history, including the presence of pre-existing eye disease and the use of glasses or contact lenses in daily life and during padel play [3] padel-related experience and exposure, including duration of practice, frequency of weekly matches, and self-assessed level of play [4] the occurrence and characteristics of padel-related injuries (i.e., both general and ocular) detailing injury mechanism, clinical diagnosis, and subsequent management; and [5] the perceived long-term impact of ocular injuries on quality of life (QoL), quality of vision (QoV), and occupational activity, as well as attitudes toward the use of protective eyewear. Respondents who indicated at the first question that they did not play padel were automatically directed to the end of the survey and were not required to complete any additional items. All information on ocular and systemic diagnoses, both pre-existing and injury-related, was self-reported by participants and not verified through medical records or clinical examination.

Padel playing level was assessed using a modified version of the International Padel Rating (IPR) scale, ranging from 1.0 to 5.0 (available at: https://www.ptrtennis.it/). Respondents were asked to identify their level according to predefined categories corresponding to beginner (1–1.5), amateur (2–2.5), intermediate (3–3.5), advanced (4–4.5), and professional (5.0). Higher IPR levels (6 and 7) were not included, as the questionnaire targeted primarily recreational and semi-professional athletes.

To assess the perceived consequences of ocular trauma, respondents were asked to rate the impact on QoL, QoV, and work activity using a 10-point numerical rating scale, ranging from 0 (no impact) to 10 (maximum impact). The scales were displayed as a graduated visual meter with fixed numeric points and color coding (green indicating lower impact and red indicating higher impact) to facilitate intuitive interpretation. For each of these domains, participants were further asked whether the reported impact was transient, defined as lasting less than one month, or long-lasting, defined as persisting beyond one month. This threshold was selected a priori to distinguish between temporary and sustained functional limitations.

The anonymous, web-based survey was distributed using a convenience sampling strategy. The survey link was disseminated via several padel-related web pages, online forums, and through social media platforms (i.e., Facebook, Instagram) commonly used by amateur and recreational players. This approach aimed to maximize reach within the target population of individuals actively engaged in the sport across Italy. Although this method does not permit the calculation of a response rate and may introduce selection bias, it is a commonly employed and pragmatic strategy for collecting self-reported data from geographically dispersed, sport-specific populations in observational survey research [11].

### Statistical analysis

All analyses were performed using R software (R for statistical computing, version 4.3.2). The unit of analysis was the individual respondent. Descriptive statistics were used to summarize demographic, geographic, and ocular characteristics of respondents. Categorical variables were reported as counts and percentages, and continuous variables were summarized as means with standard deviations (SD) and range.

To identify factors associated with ocular injury, both univariate and multivariate logistic regression analyses were conducted. In the univariate analysis, each independent variable was assessed individually for its association with the outcome (ocular injury: yes/no). Variables included match frequency, years of padel play, level of play, age (as categorical variable), gender, daily use of glasses or contact lenses, and presence of pre-existing eye disease. Odds ratios (ORs), 95% confidence intervals (CIs), and *p* values were reported.

All predictors were subsequently entered into a full multivariate logistic regression model. A backward stepwise elimination procedure based on the Akaike Information Criterion (AIC) was then applied to identify the most parsimonious model. ORs, 95% CIs, and *p* values were reported for both the full and reduced models. Multicollinearity among predictors was assessed using generalized variance inflation factors (GVIF), with values below 2 considered acceptable. Model fit was evaluated using deviance statistics and AIC values.

Statistical significance was defined as a two-sided *p* value < 0.05.

A post hoc power analysis was performed using the observed sample size and number of ocular injury events to estimate the ability of the study to detect associations of varying effect sizes in logistic regression models.

## Results

### Demographics, ocular characteristics, and padel-related experience

Of the 623 individuals who completed the survey, 583 respondents (93.7%) reported active participation in padel, while 40 (6.3%) stated they had never played the sport. Supplementary Table 1 summarizes the demographic and ocular health characteristics of the 583 padel players. The mean age was 41 years (SD = 12; range: 19-73 years). A majority identified as male (*n* = 417; 72%), with 165 participants (28%) identifying as female. Participants were drawn from all 20 Italian regions, albeit with varying representation across regions as detailed in Supplementary Table 1.

Regarding ocular health, 243 participants (42%) reported regular use of glasses or contact lenses, while 340 (58%) did not use visual aids daily. Among visual aid users (*n* = 243), contact lenses were most frequently used during padel play (*n* = 130; 53%), followed by glasses (*n* = 102; 42%), with a small minority (*n* = 11; 4.5%) reporting no use of corrective devices during matches. Pre-existing ocular pathology was reported by 99 respondents (17%). They included refractive errors (60/99, 60.6%), keratoconus (8/60, 13.3%), cataract (4/60, 6.7%), vitreoretinal disorders (4/60, 6.7%), ocular hypertension or glaucoma (3/60, 5%), maculopathies (2/60, 3.3%), and others (eg, strabismus, amblyopia, dry eye, uveitis, infectious keratitis) (18/60, 13.3%). The majority (n = 484; 83%) of the population reported no known eye disease.

Supplementary Table 2 outlines participants’ padel-related experience. Most respondents described themselves as intermediate players (*n* = 192; 33%), while in terms of years of engagement in padel, the most reported duration was 1–2 years (*n* = 196; 34%). Weekly playing frequency varied, with 216 respondents (37%) playing less than once per week and 11 individuals (2%) engaging in more than six matches weekly.

### Padel-related injuries and their impact on quality of life, visual function, and occupational activity and perceived need for ocular protection

Supplementary Table 3 summarizes the descriptive characteristics and the location (aggregated into four anatomical macro-regions) of padel-related injuries among the 583 survey respondents. Overall, 225 participants (39%) reported having sustained at least one injury during padel play. Most of these incidents were orthopedic in nature (e.g., sprains, strains, meniscal injuries, and musculoskeletal problems involving the ankle, knee, shoulder, or back) accounting for 187 reported cases. An additional 36 participants (6.2%) reported other injury types, including craniofacial trauma, superficial lacerations, tympanic membrane injuries, and trauma to the nose or teeth.

As described in Table 1, ocular injuries were reported by 60 respondents (10%). Among these, 52 individuals (87%) experienced a single ocular trauma, while 8 (13%) reported recurrent events. The predominant mechanism of injury was ball impact (n = 55; 92%), followed by trauma from the player’s own racket (*n* = 5; 8.3%). Surgical intervention was necessitated in 6 cases (10%). A large proportion of affected individuals (*n* = 43; 72%) used ophthalmic medications such as eye drops, and 10 participants (17%) missed work as a result of the ocular trauma. The most commonly self-reported ocular injuries, based on predefined survey options, were corneal abrasion (*n* = 19; 32%) and subconjunctival hemorrhage (*n* = 13; 22%), followed by macular edema (*n* = 7; 12%), vitreous detachment (*n* = 6; 10%), hematoma (*n* = 6; 10%), and retinal detachment (*n* = 5; 8.3%). Less frequent diagnoses, each reported by one respondent (1.7%), included conjunctival abrasion, iridophacodonesis, traumatic mydriasis, and anterior uveitis.

**Table 1:** Descriptive statistics and clinical details of padel-related ocular injuries reported by survey participants. Data presented as frequencies (percentages).

Ever Had Padel Injury	
* No*	358 (61%)
* Yes*	225 (39%)
Ocular Injury	
* No*	523 (90%)
* Yes*	60 (10%)
Number of Ocular Injuries (*n* = 60)	
* 1*	52 (87%)
* More than 1*	8 (13%)
Injury Mechanism (*n* = 60)	
* Ball*	55 (92%)
* Own racket*	5 (8.3%)
Ocular Surgery (*n* = 60)	
* No*	54 (90%)
* Yes*	6 (10%)
Used Eye Drops (*n* = 60)	
* No*	17 (28%)
* Yes*	43 (72%)
Missed Work (*n* = 60)	
* No*	50 (83%)
* Yes*	10 (17%)
Self -reported ocular injuries (*n* = 60)	
* Corneal abrasion*	19 (32%)
* Subconjunctival hemorrhage*	13 (22%)
* Macular edema*	7 (12%)
* Vitreous detachment*	6 (10%)
* Hematoma*	6 (10%)
* Retinal detachment*	5 (8.3%)
* Conjunctival abrasion*	1 (1.7%)
* Iridophacodonesis*	1 (1.7%)
* Traumatic mydriasis*	1 (1.7%)
* Anterior uveitis*	1 (1.7%)

The overall sample size is *N* = 583.

**Fig. 1:**
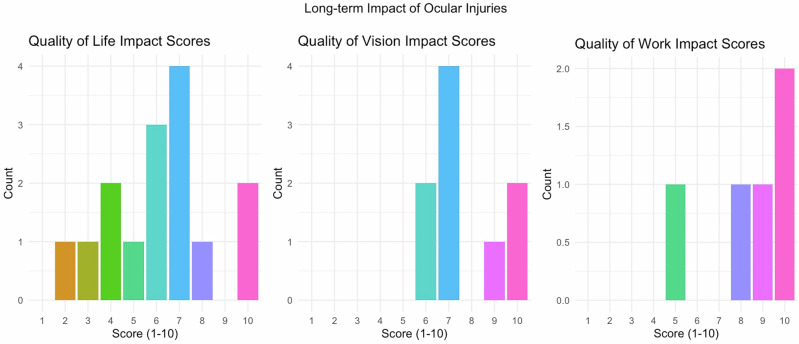
Distribution of long-term impact scores following padel-related ocular trauma. Bar charts show how injured respondents rated the lasting effect of their eye injury on a 1–10 scale (10 = maximal impairment). The upper-left panel depicts overall quality-of-life scores (14 respondents), the upper-right panel quality-of-vision scores (10 respondents), and the lower panel work-performance scores (5 respondents). Ratings for life and vision cluster mainly between 6 and 8, whereas the smaller subset reporting work impairment concentrates at the severe end of the scale [8–10].

**Fig. 2:**
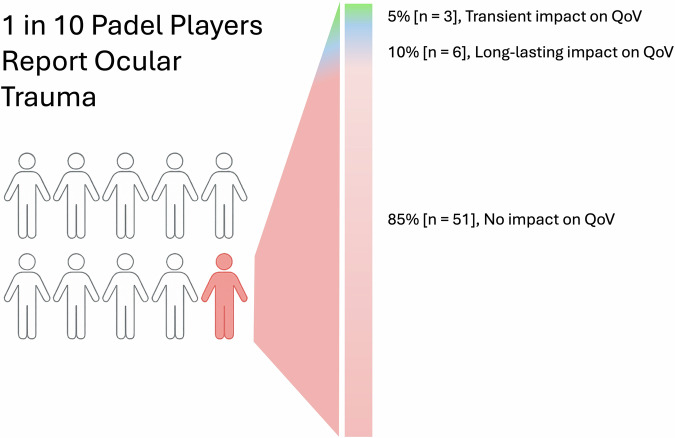
Long-term impact of padel-related ocular trauma on quality of vision. Among the 60 respondents who sustained an ocular injury, 51 (85%) reported no persistent visual impairment, 6 (10%) indicated long-lasting problems ( > 1 month) and 3 (5%) described transient symptoms that resolved within 1 month. Counts are expressed as proportions of all eye-injured players in a 100% stacked vertical bar.

Table 2 details the consequences of ocular injuries among the 60 affected participants. A negative impact QOL was reported by 15 individuals (25%), with most rating the severity as moderate to severe (scores ≥6 in 67% of cases) as shown in Fig. 1. Of those reporting QoL impairment, 6 respondents (40%) described the impact as lasting more than one month. Similarly, 9 participants (15%) reported a reduction in QoV, with the majority assigning scores of ≥7 (*n* = 7; 78%). As shown in Fig. 2, long-lasting impact on vision ( > 1 month) was noted by 6 respondents (67%) in this subgroup. Occupational functioning was affected in 5 cases (8.3%), with 3 participants (60%) experiencing long-term work-related limitations.

**Table 2:** Impact of ocular injuries on quality of life, quality of vision, and occupational functioning among respondents reporting padel-related ocular injuries.

Impact on quality of life (QOL)	
* No*	45 (75%)
* Yes*	15 (25%)
QOL Impact Score (0–10) (*n* = 15)	
* 1*	0 (0%)
* 2*	1 (6.7%)
* 3*	1 (6.7%)
* 4*	2 (13%)
* 5*	1 (6.7%)
* 6*	3 (20%)
* 7*	4 (27%)
* 8*	1 (6.7%)
* 10*	2 (13%)
QOL impact level (*n* = 15)	
* Long-lasting (>1 month)*	6 (40%)
* Transient (<1 month)*	9 (60%)
Impact on quality of vision (QOV)	
* No*	51 (85%)
* Yes*	9 (15%)
QOV impact score (0–10) (*n* = 9)	
* 1*	0 (0%)
* 2*	0 (0%)
* 3*	0 (0%)
* 4*	0 (0%)
* 5*	0 (0%)
* 6*	2 (22%)
* 7*	4 (44%)
* 9*	1 (11%)
* 10*	2 (22%)
QOV Impact Level (*n* = 9)	
* Long-lasting (>1 month)*	6 (67%)
* Transient (<1 month)*	3 (33%)
Impact on Work	
* No*	55 (92%)
* Yes*	5 (8.3%)
Work Impact Score (0–10) (*n* = 5)	
* 1*	0 (0%)
* 2*	0 (0%)
* 3*	0 (0%)
* 4*	0 (0%)
* 5*	1 (20%)
* 6*	0 (0%)
* 7*	0 (0%)
* 8*	1 (20%)
* 9*	1 (20%)
* 10*	2 (40%)
Work Impact Level (n = 5)	
* Long-lasting (>1 month)*	3 (60%)
* Transient (<1 month)*	2 (40%)

Data presented as frequencies (percentages).

**Fig. 3:**
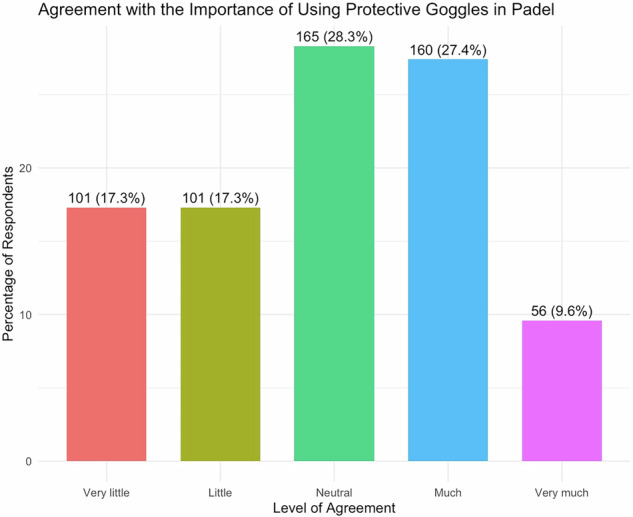
Attitudes toward protective eye equipment adoptions among active padel players. Distribution of responses from 583 Italian athletes to a five-point Likert item assessing agreement with the importance of wearing protective eyewear while playing. “Very little” and “Little” agreement together account for 202 respondents (34.6%), “Neutral” for 165 (28.3%), “Much” for 160 (27.4%), and “Very much” for only 56 (9.6%), indicating limited endorsement of routine goggle use despite the documented injury risk.

Figure 3 illustrates respondents’ perspectives on the importance of protective eyewear in padel. Attitudes were heterogeneous: 56 individuals (9.6%) strongly agreed that protective goggles are important, and 160 (27%) expressed moderate agreement. In contrast, 202 respondents (35%) exhibited low concern, equally split between those indicating “very little” (*n* = 101) and “little” (*n* = 101) agreement. Additionally, 165 participants (28%) reported a neutral or indifferent attitude.

When stratified by ocular injury history, attitudes toward protective eyewear differed significantly. Respondents with a history of self-reported ocular trauma were more likely to strongly endorse the importance of protective goggles compared with non-injured players (32% vs 7.1%), whereas indifference was more common among those without prior injury (30% vs 12%) (Pearson’s α ^2^, *p* < 0.001). Overall, these findings indicate that although prior injury experience appears to increase support for eye protection, a substantial proportion of players remain disengaged or unconvinced of its necessity.

### Risk factor analysis for padel-related injury

The results of both univariate and multivariate logistic regression analyses evaluating potential predictors of ocular injury among padel players are presented in Supplementary Tables 4, 5.

#### Univariate analysis

In univariate analyses, a higher frequency of weekly match play emerged as a strong predictor of ocular trauma. Specifically, engaging in 4–6 matches per week was associated with a markedly increased risk (OR 37.5; 95% CI 12.7–139.0; *p* < 0.001), followed by 7–8 matches (OR 11.8; 95% CI 1.50–69.4; p = 0.008) and 1–3 matches (OR 7.05; 95% CI 2.78–23.8; *p* < 0.001).

Longer duration of padel practice also demonstrated significant associations, with increased odds observed among those playing for more than six years (OR 3.27; 95% CI 1.25–8.88; *p* = 0.016) and for 3–5 years (OR 2.51; 95% CI 1.15–6.11; *p* = 0.029), compared to those with shorter exposure.

Playing level was likewise significantly associated with risk: intermediate (OR 12.8; 95% CI 2.63–231.0; *p* = 0.013), advanced (OR 17.9; 95% CI 3.66–323.0; *p* = 0.005), and professional players (OR 20.1; 95% CI 3.26–389.0; *p* = 0.006) exhibited substantially elevated odds relative to beginners. Age was another relevant factor, with players aged 40–50 years (OR 3.47; 95% CI 1.43–9.74; *p* = 0.010) and >50 years (OR 2.61; 95% CI 1.06–7.35; *p* = 0.048) showing significantly increased injury risk compared to younger counterparts.

No statistically significant associations were identified for gender, daily use of visual aids, or a history of ocular pathology.

#### Multivariable models

In the full multivariate model, the frequency of weekly play remained the most robust independent predictor of ocular injury. Statistically significant associations were retained for playing 1–3 matches per week (OR 4.38; 95% CI 1.59–15.60; *p* = 0.009), 4–6 matches (OR 26.85; 95% CI 7.98-110.25; *p* < 0.001), and 7–8 matches (OR 9.06; 95% CI 1.02–62.53; *p* = 0.029), suggesting a dose-response relationship between playing frequency and injury risk. Although the directionality of associations for age and level of play remained consistent with univariate findings, these variables did not achieve statistical significance in the multivariable context, potentially due to reduced precision or residual confounding. Male gender was inversely associated with injury risk (–0.66; OR 0.51; 95% CI 0.27–1.01; *p* = 0.049), suggestive of a potential protective effect, though the finding approached the threshold for statistical significance.

Backward stepwise elimination based on AIC identified a more parsimonious model retaining three variables: match frequency, daily use of corrective eyewear, and pre-existing ocular pathology. Match frequency remained highly predictive, with ORs of 7.01 (95% CI 2.75–23.71; *p* < 0.001) for 1–3 matches, 38.17 (95% CI 12.80–142.47; *p* < 0.001) for 4–6 matches, and 10.92 (95% CI 1.37–65.08; *p* = 0.011) for 7–8 matches per week. Use of daily glasses (OR 0.60; 95% CI 0.31–1.09; *p* = 0.102) and a history of ocular pathology (OR 1.73; 95% CI 0.80–3.51; *p* = 0.144) did not reach statistical significance yet were retained based on model fit criteria.

Collectively, these findings identify frequency of padel match play as the most consistent and robust predictor of ocular injury. Although other variables (e.g., playing experience, skill level, age, and ocular comorbidities) may contribute to injury risk, their influence appears to be attenuated when adjusted for weekly playing intensity.

### Post-hoc power calculation analysis

A post-hoc power analysis was conducted to determine the sensitivity of the survey to detect associations between risk factors and the occurrence of ocular injury among padel players. Based on the observed sample size (*n* = 583), with 60 cases (10%) reporting at least one ocular injury and 523 controls, the analysis revealed that the study had 84.5% power to detect an OR of 1.5 at a significance level of α = 0.05. Power increased progressively with larger effect sizes, exceeding 90% for an OR of 1.6, and reaching virtually 100% for ORs ≥2.1. These findings suggest that the survey was well powered to detect moderate to large associations between exposure variables (e.g., frequency of matches) and ocular injury risk.

## Discussion

The results of this first investigation on self-reported ocular injuries in padel, based on responses from 583 participants across various Italian regions (albeit with unequal regional representation), provide preliminary insights into the perceived burden and characteristic of ocular trauma in this sport. In a cohort with a mean age of 41 years, a predominantly male composition, and a heterogeneous distribution of self-reported playing levels, approximately 10% of respondents reported having experienced at least one ocular injury. Notably, in 25% of these cases, the injury had long-term consequences on QoL, QoV, and, to a lesser extent, occupational function. To contextualize these findings, it is worth noting that the estimated risk of visual loss following modern cataract surgery—an elective procedure performed under controlled clinical conditions—is fewer than 1 in 200 patients [12–14]. This comparison provides contextual perspective on the potential burden of ocular trauma in padel and underscore the relevance for targeted preventive strategies in this rapidly growing sport.

Of particular relevance was the strong and independent association between weekly match frequency and the likelihood of sustaining ocular trauma. This dose-response relationship persisted across univariate and multivariable models, with adjusted OR as high as 38 for those playing four to six matches per week compared to less frequent players. These results suggest that cumulative exposure and playing intensity may play an important role in ocular injury risk in padel. Although longer playing history, higher skill levels, and older age also showed associations with injury risk in univariate analyses, their effects were attenuated in the adjusted models, suggesting potential confounding.

Interestingly, male gender was inversely associated with ocular injury risk in the full multivariable model, although this finding approached but did not cross the threshold for statistical significance. Likewise, daily use of corrective eyewear and pre-existing ocular pathology were retained in the reduced model based on AIC yet did not demonstrate independent predictive value. These observations may inform future research exploring the complex interplay between visual health, uncorrected refractive error, and trauma susceptibility in padel.

Our findings are supported by and aligned with prior literature. For instance, Kasiga et al.‘s Swedish study on 255 patients with sports-related ocular trauma identified padel as the leading cause of ocular injury during the most recent observation year [1]. Additionally, Merino et al.‘s case series of 34 patients with posterior segment ocular injuries related to padel highlights the potentially severe consequences of such trauma [15] Although most subjects in their study achieved satisfactory visual recovery, several experienced serious complications including retinal detachment and angle recession glaucoma [15] Our findings corroborate these concerns: although the proportion of severe cases was smaller, events such as retinal detachment, macular edema, and hemorrhages were reported, and 6 of 60 affected individuals (10%) required surgical treatment.

While the eye appears as a particularly vulnerable anatomical site in padel, musculoskeletal injuries—such as sprains, strains, and meniscal injuries—remained the most frequently reported in our survey (*n* = 187), underscoring the multifaceted nature of injury risk in this sport. Two Spanish studies involving 60 [16] and 131[4] padel players respectively identified high rates of elbow tendinopathy, back pain, and knee injuries, influenced by age, BMI, and training frequency. Similar findings were reported by Muñoz et al., who, in a survey involving 950 participants, identified weekly training volume, player experience, and gender as key factors to consider in the prevention of injuries among amateur padel players [3] A recent systematic review analyzing 8 studies with a cumulative total of 2022 participants estimated a general injury incidence of approximately 3 injuries per 1000 h of training and 8 injuries per 1000 matches [2]. These figures predominantly reflect musculoskeletal trauma and are consistent with our dataset, considering that 48% of respondents played 1–3 matches weekly. However, our study emphasizes ocular trauma—a category often underrepresented in broader reviews—highlighting its importance for player safety.

Previous literature identifies several contributing factors to injury risk, including increased exposure due to frequent play, overuse, and greater probability of high-intensity impacts. These patterns emphasize the need for targeted prevention strategies, especially for advanced-level or high-frequency players. Despite the reported impact of ocular trauma, up to 60% of respondents expressed little or no interest in the use of protective eyewear. Importantly, attitudes differed significantly according to injury history: players who had experienced self-reported ocular trauma were substantially more likely to strongly endorse protective goggles than those without prior injury. This pattern suggests that direct injury experience may increase perceived vulnerability and support for preventive strategies, whereas players without prior events may underestimate their personal risk. From a preventive perspective, this highlights a critical need to increase awareness of ocular trauma risks in padel before injury occurs. The neutral attitude of many players may represent an opportunity: targeted educational campaigns could shift attitudes toward more widespread adoption of protective devices. Simultaneously, gaining insight into the barriers to adoption—such as discomfort, visual interference, or perceived inconvenience–could help inform the design of more user-friendly and sport-specific protective solutions.

An additional consideration relates to the regulatory and standardization framework surrounding protective eyewear in racket sports. At present, no padel-specific international standard defines performance requirements for eye protection. In Europe, ISO 18527-2 [17] and in the United States, ASTM F3164 [18] define performance standards for eye protectors in selected racket sports, specifying impact resistance, optical quality, and coverage criteria for high-velocity ball sports. Although padel has distinct court characteristics, these standards may represent a reasonable minimum benchmark in the absence of sport-specific requirements. Importantly, prevention strategies extend beyond technical specifications. In Europe, protective sports eyewear falls within the scope of Regulation (EU) 2016/425 on Personal Protective Equipment [19]. Yet regulatory compliance alone does not ensure uptake. Education of players, coaches, and clubs, together with potential recommendations from sporting federations may be central to improving adoption, as observed in other racket sports such as squash [20].

Future research should build on these findings through prospective, clinically validated surveillance systems capable of capturing confirmed diagnoses and standardized exposure metrics. National emergency department registry analyses in comparable racket sports, such as recent NEISS-based investigations of pickleball-related ocular trauma, have demonstrated the feasibility and value of weighted population-level injury monitoring [7, 8]. Similar structured surveillance approaches for padel, ideally integrating ophthalmic confirmation of diagnoses and standardized injury classification, would allow more accurate estimation of incidence, severity distribution, and long-term visual outcomes. Multicenter collaboration across European countries, where padel participation is expanding rapidly, may be particularly informative. In parallel, interventional studies evaluating the effectiveness, acceptability, and performance standards of sport-specific protective eyewear in padel are warranted.

### Strengths and limitations

Among the strengths of the study are its large sample size and specific focus on ocular trauma, an area that remains underexplored in the existing sports and eye medicine literature.

However, some limitations may hamper the reliability of the proposed results. Several effect estimates, especially those for the highest playing-frequency categories and for advanced or professional skill levels, are accompanied by wide 95%CIs, likely reflecting sparse data within certain strata and heterogeneity in self-reported exposures. Although the direction of associations was consistent across analyses and supported by adequate statistical power, the reliance on self-reported data introduces potential recall bias and inaccurate estimation of injury severity. In particular, ocular diagnoses (i.e., both pre-existing conditions and padel-related injuries) were reported by respondents through a structured questionnaire rather than confirmed by ophthalmic examination, raising the possibility of misclassification or misunderstanding of medical terminology (e.g., “macular edema” or “retinal detachment”). Moreover, although the questionnaire underwent internal review and pilot testing, it did not undergo formal external validation, and variability in respondents’ health literacy may have further influenced the accuracy of self-reported clinical information.

Participation was voluntary and the recruitment relied on convenience sampling; therefore, self-selection bias cannot be excluded. Respondents with prior ocular trauma or heightened awareness of eye health may have been more likely to complete the survey, likely inflating the observed proportions. Accordingly, the reported estimates should be interpreted as self-reported frequencies among respondents rather than population-level prevalence or incidence measures.

Additionally, the predefined mechanism categories focused on ball, racket, and wall/net impacts and did not include a dedicated option for direct player-to-player bodily contact (e.g., finger or elbow), which may have led to under-ascertainment of this potential mechanism

Finally, the cross-sectional design precludes any inference on causality.

## Conclusions

Based on data from 583 respondents to a convenience-based survey distributed nationally across Italy, this study provides insights into the self-reported burden, risk factors, and perceived consequences of ocular injuries in padel. While musculoskeletal trauma remained more frequent overall, the eye emerged as a vulnerable site for potentially vision-threatening injury, particularly among players with high match frequency. Importantly, despite reported impacts on quality of life and vision, protective eyewear use was limited. These findings should be considered hypothesis-generating, underscoring the need for prospective, clinically verified studies and for public health initiatives to promote preventive strategies in this rapidly expanding sport.

## Summary

### What was known before

- Padel participation is rising rapidly; most published injuries are musculoskeletal. Ocular trauma is recognised in racket sports but remains under-reported in padel.
- High-velocity play in confined courts makes ball or racket impact plausible mechanisms of eye injury.
- Robust epidemiology on frequency, risk factors, and perceived need for protective eyewear in padel has been lacking.

### What this study adds

- In a cross-sectional survey of 583 active players (May 2022–March 2025), 10.3% reported ocular trauma, predominantly from ball impact, with measurable effects on QoL, visual quality, work function, and a 10% surgical rate among cases.
- Weekly playing frequency is an independent predictor of ocular injury (e.g., 4–6 matches/week OR = 26.9), whereas other factors lose significance after adjustment.
- Acceptance of protective eyewear is low despite risk, highlighting the need for targeted education and more acceptable protective designs; supplementary analyses also profile non-ocular injury sites to inform prevention.

## Supplementary information

Supplemental Data

## Data Availability

The datasets generated and analyzed during the current study are available from the corresponding author on reasonable request.
