# Supplementary material for: Ocular injuries in padel: findings from a survey on frequency, risk factors, and perceptions toward protective eyewear
Source: Eye (Lond). 2026 Apr 20;40(10):1469–76. doi: 10.1038/s41433-026-04447-8 (PMC13341748; doi:10.1038/s41433-026-04447-8)
Supplement: Supplementary file 1 — Supplemental Data [file 41433_2026_4447_MOESM1_ESM.docx]

**Supplemental File 1.**

**Survey Questionnaire: Italian and English Versions (Complete)**

**Introduction / Introduzione**

**Italiano:**
Il seguente questionario che ti invitiamo a compilare è stato redatto da medici oculisti al fine di studiare il rischio di traumi oculari accidentali avvenuti durante la pratica agonistica e non agonistica del gioco del padel.

**English:**
The following questionnaire, which we invite you to complete, was developed by ophthalmologists to study the risk of accidental ocular trauma occurring during competitive and non-competitive padel play.

**Section 1 – Screening**

**1. Giochi mai a padel?**
(Do you play padel?)

- Sì (Yes) → Proceed to question 2
- No (No) → Skip to the end of the questionnaire

**Section 2 – Demographics**

**2. Quanti anni hai?**
(How old are you?)

**3. Genere:**
(Gender)

- Maschile (Male)
- Femminile (Female)
- Preferisco non specificare (Prefer not to say)

**4. In quale regione vivi?**
(Which region do you live in?)
[List of 20 Italian regions]

**5. In quale città vivi?**
(Which city do you live in?)

**Section 3 – Ocular Health**

**6. Utilizzi occhiali o lenti a contatto nella vita di tutti i giorni?**
(Do you wear glasses or contact lenses in daily life?)

**7. Quando giochi a padel, utilizzi:**
(When playing padel, do you use:)

- Occhiali (Glasses)
- Lenti a contatto (Contact lenses)
- Nessuno (None)

**8. Ti è mai stata diagnosticata una patologia oculare?**
(Have you ever been diagnosed with an eye condition?)

- Sì → Specificare (Yes → Please specify)
- No (No)

**Section 4 – Padel Experience**

**9. Sulla base della scala di riferimento, potresti definire il tuo livello di gioco?**
(According to the reference scale, how would you define your level of play?)

- 1–1.5: Principiante (Beginner)
- 2–2.5: Amatore (Amateur)
- 3–3.5: Intermedio (Intermediate)
- 4–4.5: Avanzato (Advanced)
- 5: Pro (Professional)

**10. Da quanto tempo giochi a padel?**
(How long have you been playing padel?)

- <1 anno (<1 year)
- 1–2 anni (1–2 years)
- 3–5 anni (3–5 years)
- 5 anni (>5 years)

**11. Quante partite di padel giochi ogni settimana?**
(How many padel matches do you play per week?)

- <1
- 1–3
- 4–6
- 6

**Section 5 – Injury History**

**12. Hai mai subito un infortunio giocando a padel?**
(Have you ever sustained an injury while playing padel?)

- Sì (Yes) → Proceed to question 13
- No (No) → Proceed to question 15

**13. In quale distretto corporeo?**
(Which body region?)

- Caviglia (Ankle)
- Ginocchio (Knee)
- Anca (Hip)
- Schiena (Back)
- Polso (Wrist)
- Gomito (Elbow)
- Spalla (Shoulder)
- Collo (Neck)
- Testa (Head)

**14. Hai mai subito un infortunio agli occhi giocando a padel?**
(Have you ever sustained an eye injury while playing padel?)

- Sì (Yes)
- No (No)

**15. Quanti infortuni oculari hai avuto?**
(How many ocular injuries have you had?)

- 1
- Più di 1 (More than 1)

**16. L'infortunio è avvenuto con:**
(The injury was caused by:)

- Pallina (Ball)
- Racchetta propria (Own racket)
- Racchetta di un altro giocatore (Other player’s racket)
- Parete/Rete (Wall/Net)

**17. Dopo l'infortunio, cosa hai fatto?**
(After the injury, what did you do?)

- Pronto soccorso (Visited the ER)
- Medico/oculista nei giorni successivi (Doctor/ophthalmologist in following days)

**18. Quale diagnosi ti è stata formulata?**
(What was the diagnosis?)

- Emorragia sottocongiuntivale (Subconjunctival hemorrhage)
- Abrasione corneale (Corneal abrasion)
- Cataratta traumatica (Traumatic cataract)
- Edema maculare (Macular edema)
- Distacco di retina (Retinal detachment)
- Distacco di vitreo (Vitreous detachment)
- Altro (Other)

**19. Sei stato sottoposto ad intervento chirurgico?**
(Did you undergo eye surgery?)

**20. Hai usato colliri nei giorni successivi?**
(Did you use eye drops in the following days?)

**21. Hai perso giornate lavorative?**
(Did you miss work due to the injury?)

**Section 6 – Impact Assessment**

**22. Il trauma oculare ha avuto un impatto sulla tua qualità della vita (QoL)?**
(Did the eye injury impact your quality of life?)

**23. Indica su una scala da 1 a 10 l'entità dell'impatto sulla QoL.**
(Rate the impact on QoL on a scale from 1 to 10.)

**24. L'impatto è stato:**
(The impact was:)

- Transitorio (<1 mese) (Transient <1 month)
- Permanente (>1 mese) (Permanent >1 month)

**25. Il trauma ha avuto un impatto sulla tua qualità della vista (QoV)?**
(Did the injury impact your quality of vision?)

**26. Indica su una scala da 1 a 10 l'entità dell'impatto sulla QoV.**
(Rate the impact on QoV on a scale from 1 to 10.)

**27. L'impatto è stato:**
(The impact was:)

- Transitorio (<1 mese) (Transient <1 month)
- Permanente (>1 mese) (Permanent >1 month)

**28. Il trauma ha avuto un impatto sulla tua attività lavorativa?**
(Did the injury impact your work activity?)

**29. Indica su una scala da 1 a 10 l'entità dell'impatto lavorativo.**
(Rate the impact on your work activity on a scale from 1 to 10.)

**30. L'impatto è stato:**
(The impact was:)

- Transitorio (<1 mese) (Transient <1 month)
- Permanente (>1 mese) (Permanent >1 month)

**Section 7 – Attitudes Toward Protection**

**31. Saresti favorevole all'utilizzo di occhiali protettivi durante il gioco del padel?**
(Would you be in favor of wearing protective eyewear during padel?)

- Molto poco (Very little)
- Poco (Little)
- Indifferente (Indifferent)
- Molto (Much)
- Moltissimo (Very much)

**Grazie per il tuo prezioso aiuto!**
**Thank you for your valuable contribution!**

**Supplemental Table 1**. **Demographic and geographic characteristics of survey respondents reporting active participation in padel (N = 583).** Data presented as mean (standard deviation) with range for age, and as frequencies (percentages) for gender and regional distribution. (Abbreviations: CL, contact lenses; SD, standard deviation).

| Variable | n (%) or Mean (SD) [Range] |
| --- | --- |
| Age (years) | 41 (12) [19–73] |
| Gender |  |
| *Female* | 165 (28%) |
| *Male* | 417 (72%) |
| *Prefer not to specify* | 1 (0.2%) |
| Region |  |
| *Abruzzo* | 7 (1.2%) |
| *Basilicata* | 5 (0.9%) |
| *Calabria* | 2 (0.3%) |
| *Campania* | 41 (7.0%) |
| *Emilia-Romagna* | 17 (2.9%) |
| *Lazio* | 363 (62.0%) |
| *Liguria* | 4 (0.7%) |
| *Lombardia* | 28 (4.8%) |
| *Marche* | 1 (0.2%) |
| *Molise* | 6 (1.0%) |
| *Piemonte* | 10 (1.7%) |
| *Puglia* | 39 (6.7%) |
| *Sardegna* | 4 (0.7%) |
| *Sicilia* | 6 (1.0%) |
| *Toscana* | 18 (3.1%) |
| *Umbria* | 3 (0.5%) |
| *Veneto* | 29 (5.0%) |
| Daily Glasses/CL Use (n = 583) |  |
| *No* | 340 (58%) |
| *Yes* | 243 (42%) |
| Glasses/CL When Playing Padel (n=243) |  |
| *Contact lenses* | 130 (53%) |
| *Glasses* | 102 (42%) |
| *None* | 11 (4.5%) |
| Pre-existing Eye Pathology |  |
| *No* | 484 (83%) |
| *Yes* | 99 (17%) |

**Supplemental Table 2.** Summary of respondents' padel experience including self-reported playing level, duration of participation, and weekly frequency of matches played. Data are presented as counts (percentages). (Abbreviations. N = number of matches).

| Matches per Week (n) | |
| --- | --- |
| *< 1* | 216 (37%) |
| *1 - 3 matches* | 315 (54%) |
| *4 - 6 matches* | 41 (7%) |
| *7 - 8 matches* | 11 (2%) |
| Duration of Play | |
| *< 1 year* | 134 (23%) |
| *1 - 2 years* | 196 (34%) |
| *3 - 5 years* | 189 (32%) |
| *> 6 years* | 64 (11%) |
| Level of Play | |
| *1–1.5 (Beginner)* | 95 (16%) |
| *2–2.5 (Amateur)* | 118 (20%) |
| *3–3.5 (Intermediate)* | 192 (33%) |
| *4–4.5 (Advanced)* | 144 (25%) |
| *5 (Professional)* | 34 (6%) |

**Supplemental Table 3.** Frequency of self-reported trauma locations among padel players, aggregated into four anatomical macro-regions (head, trunk, upper extremities, lower extremities). Counts represent the number of times a body district was mentioned across questionnaire responses. Injuries to the eye/periocular region were deliberately excluded from this analysis. Because respondents could report injuries in more than one body district, summed counts exceed the number of affected individuals (n = 225).

| Macro-region | Districts counted in the group | n (mentions) |
| --- | --- | --- |
| Head | head/face, teeth/dental, nose, ear/tympanum | 36 |
| Trunk | back/lumbar, neck/cervical, generic tendon/soft-tissue torso | 46 |
| Upper extremities | shoulder, elbow, wrist, hand, finger/phalanx | 97 |
| Lower extremities | hip, buttock/gluteal, groin/adductor, thigh, knee/meniscus, calf/gastrocnemius, leg-muscle, ankle, heel, foot | 134 |
| Total (non-ocular) | – | 313 |

| Variable | Level | OR | 95% CI Lower | 95% CI Upper | p-value |
| --- | --- | --- | --- | --- | --- |
| Matches per week | < 1 | - | - | - | - |
|  | 1 - 3 matches | 7.05 | 2.78 | 23.83 | **<0.001** |
|  | 4 - 6 matches | 37.54 | 12.73 | 138.95 | **<0.001** |
|  | 7 - 8 matches | 11.78 | 1.50 | 69.44 | **0.008** |
| Duration of play | < 1 year | - | - | - | - |
|  | 1 - 2 years | 3.27 | 1.25 | 8.88 | **0.016** |
|  | 3 - 5 years | 1.31 | 0.55 | 3.33 | 0.556 |
|  | > 6 years | 2.51 | 1.15 | 6.11 | **0.029** |
| Level of play | Beginner (1 – 1.5) | - | - | - | - |
|  | Amateur (2–2.5) | 5.93 | 1.03 | 111.84 | 0.099 |
|  | Intermediate (3–3.5) | 12.79 | 2.63 | 230.70 | **0.013** |
|  | Advanced (4–4.5) | 17.87 | 3.66 | 322.57 | **0.005** |
|  | Professional (5) | 20.14 | 3.26 | 388.99 | **0.006** |
| Age category | < 30 years | - | - | - | - |
|  | 30–40 years | 1.82 | 0.71 | 5.26 | 0.236 |
|  | 40–50 years | 3.47 | 1.43 | 9.74 | **0.010** |
|  | 50+ years | 2.61 | 1.06 | 7.35 | **0.048** |
| Gender | Female | - | - | - | - |
|  | Male | 0.84 | 0.48 | 1.52 | 0.548 |
|  | Prefer not to say | 0.00 | NA | NA | 0.989 |
| Daily use of CLs or Glasses | No | - | - | - | - |
|  | Yes | 0.57 | 0.31 | 1.00 | 0.055 |
| Coexistent eye disease | No | - | - | - | - |
|  | Yes | 1.25 | 0.61 | 2.38 | 0.512 |

**Supplemental Table 4.** Univariable logistic regression analysis assessing associations between participant characteristics and the risk of experiencing padel-related ocular injury. Odds ratios (ORs), 95% confidence intervals (CIs), and p-values are reported for each predictor variable, using the indicated reference categories. Statistically significant associations (p < 0.05) are shown in bold. Analyses were conducted using the individual respondent as the unit of analysis. (Abbreviations: CLs, contact lenses).

**Supplemental Table 5.** Multivariate logistic regression models assessing the association between participant characteristics and the risk of padel-related ocular injury. The table reports odds ratios (OR), 95% confidence intervals (CI), and p-values for each level of the included variables. The full model includes all predictors, while the reduced model was derived through backward stepwise elimination based on Akaike Information Criterion criteria. Statistically significant associations (p < 0.05) are highlighted in bold. Values are referenced against the baseline category within each variable.

| Variable | Level | OR | 95% CI Lower | 95% CI Upper | p-value |
| --- | --- | --- | --- | --- | --- |
| Full model | | | | | |
| Matches per week | < 1 | - | - | - | - |
|  | 1 - 3 matches | 4.38 | 1.59 | 15.6 | **0.009** |
|  | 4 - 6 matches | 26.85 | 7.98 | 110.25 | **<0.001** |
|  | 7 - 8 matches | 9.06 | 1.02 | 62.53 | **0.029** |
| Duration of play | < 1 year | - | - | - | - |
|  | 1 - 2 years | 0.54 | 0.17 | 1.71 | 0.283 |
|  | 3 - 5 years | 0.71 | 0.22 | 2.39 | 0.573 |
|  | > 6 years | 0.87 | 0.21 | 3.65 | 0.849 |
| Level of play | Beginner (1 – 1.5) | - | - | - | - |
|  | Amateur (2–2.5) | 4.86 | 0.73 | 97.29 | 0.163 |
|  | Intermediate (3–3.5) | 8.65 | 1.22 | 181.76 | 0.066 |
|  | Advanced (4–4.5) | 7.14 | 0.91 | 157.25 | 0.106 |
|  | Professional (5) | 5.55 | 0.53 | 136.63 | 0.193 |
| Age category | < 30 years | - | - | - | - |
|  | 30–40 years | 1.56 | 0.55 | 4.89 | 0.415 |
|  | 40–50 years | 1.76 | 0.65 | 5.37 | 0.289 |
|  | 50+ years | 1.76 | 0.63 | 5.48 | 0.298 |
| Gender | Female | - | - | - | - |
|  | Male | 0.51 | 0.27 | 1.01 | **0.049** |
|  | Prefer not to say | 0 | NA | NA | 0.99 |
| Daily use of CL or Glasses | No | - | - | - | - |
|  | Yes | 0.59 | 0.3 | 1.11 | 0.112 |
| Coexistent eye disease | No | - | - | - | - |
|  | Yes | 1.74 | 0.8 | 3.62 | 0.147 |
| Reduced Model | | | | | |
| Matches per week | < 1 | NA | NA | NA | NA |
|  | 1 - 3 matches | 7.01 | 2.75 | 23.71 | **0.0003** |
|  | 4 - 6 matches | 38.17 | 12.8 | 142.47 | **<0.001** |
|  | 7 - 8 matches | 10.92 | 1.37 | 65.08 | **0.0107** |
| Daily use of CL or Glasses | No | - | - | - | - |
|  | Yes | 0.6 | 0.31 | 1.09 | 0.102 |
| Coexistent eye disease | No | - | - | - | - |
|  | Yes | 1.73 | 0.8 | 3.51 | 0.144 |
